# Spatial Memory Engram in the Mouse Retrosplenial Cortex

**DOI:** 10.1016/j.cub.2018.05.002

**Published:** 2018-06-18

**Authors:** Michal M. Milczarek, Seralynne D. Vann, Frank Sengpiel

**Affiliations:** 1 School of Psychology, Cardiff University, Cardiff, UK; 2 School of Biosciences, Cardiff University, Cardiff, UK

**Keywords:** c-Fos, immediate-early gene, reference memory, 2-photon imaging, retrosplenial cortex, mouse, engram, radial-arm maze

## Abstract

Memory relies on lasting adaptations of neuronal properties elicited by stimulus-driven plastic changes [1]. The strengthening (and weakening) of synapses results in the establishment of functional ensembles. It is presumed that such ensembles (or engrams) are activated during memory acquisition and re-activated upon memory retrieval. The retrosplenial cortex (RSC) has emerged as a key brain area supporting memory [2], including episodic and topographical memory in humans [3, 4, 5], as well as spatial memory in rodents [6, 7]. Dysgranular RSC is densely connected with dorsal stream visual areas [8] and contains place-like and head-direction cells, making it a prime candidate for integrating navigational information [9]. While previous reports [6, 10] describe the recruitment of RSC ensembles during navigational tasks, such ensembles have never been tracked long enough to provide evidence of stable engrams and have not been related to the retention of long-term memory. Here, we used *in vivo* 2-photon imaging to analyze patterns of activity of over 6,000 neurons within dysgranular RSC. Eight mice were trained on a spatial memory task. Learning was accompanied by the gradual emergence of a context-specific pattern of neuronal activity over a 3-week period, which was re-instated upon retrieval more than 3 weeks later. The stability of this memory engram was predictive of the degree of forgetting; more stable engrams were associated with better performance. This provides direct evidence for the interdependence of spatial memory consolidation and RSC engram formation. Our results demonstrate the participation of RSC in spatial memory storage at the level of neuronal ensembles.

## Highlights

• Longitudinal *C-fos* imaging reveals retrosplenial spatial memory engrams in mice
• Engrams become progressively more stable with learning and are maintained over weeks
• The degree of memory retention is related to the stability of the engrams

## Teaser

Milczarek et al. identify spatial memory engrams in the mouse retrosplenial cortex using *in vivo C-fos* imaging. The engrams become progressively more stable with learning and are maintained over several weeks. The degree of memory retention is related to the stability of the engrams, highlighting a possible mechanism for memory consolidation.

## Results and Discussion

### *In Vivo* Immediate-Early Gene Imaging

**Figure 1:**
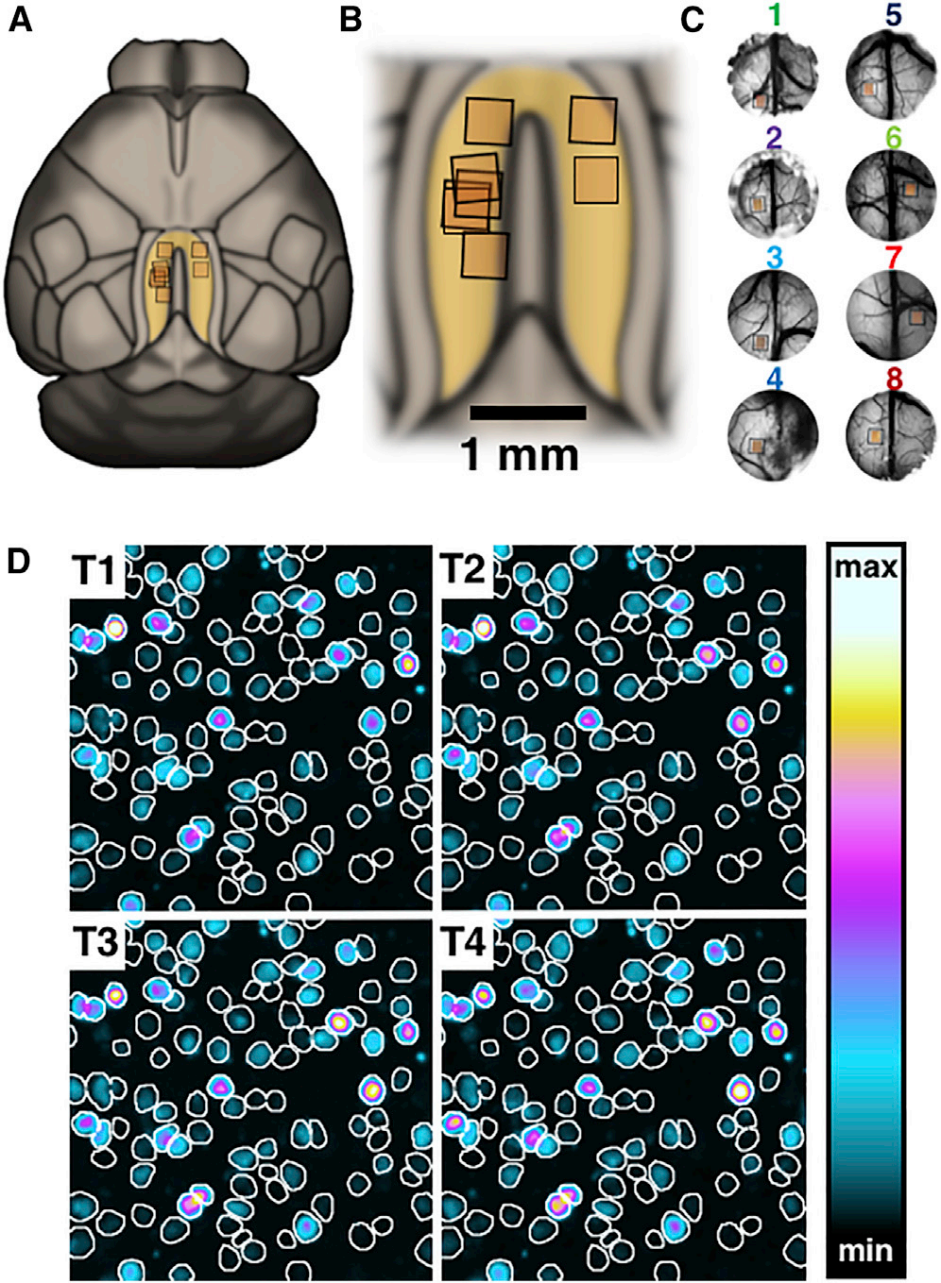
*In Vivo* Immediate-Early Gene Imaging (A–C) Regions of interest (ROIs) selected for 2-photon imaging. (A) Representation of the top view of the mouse brain based on the Allen Institute’s 3D mouse brain atlas with the positions of the ROIs. The dorsal (dysgranular) retrosplenial cortex (RSC) is highlighted in yellow. (B) A zoomed-in image showing the RSC. The images in (C) are brightfield photographs of the craniotomy areas in each animal. The numbering of cases and associated color-code is carried forward to subsequent figures. (D) Task-evoked change in fluorescence. The white outlines denote the positions of cells detected across the whole study, while the coloring indicates the level of fluorescence observed at four timepoints following exposure to the maze on a single session. The intervals are 1.5–2 hr (T1), 2–2.5 hr (T2), 2.5–3 hr (T3), and 3–3.5 hr (T4) from the beginning of the task, respectively. See also Figure S1.

We employed mice expressing a short-lived version of the enhanced green fluorescent protein (eGFP) under the control of the *C-fos* gene promoter [11], reportedly restricted to excitatory neurons [12, 13]. The short half-life of the protein, as well as the fact that it is not a conjugate of c-Fos, makes it ideal for *in vivo* tracking of immediate-early gene expression across multiple imaging sessions. We confirmed the identity of eGFP-expressing cells as c-Fos-positive neurons in postmortem examination of tissue (Figure S1A). Cranial windows were implanted over the dorsal (dysgranular) RSC of eight mice (Figures 1A–1C) to examine the relationship between the acquisition of a reference memory task in the radial-arm maze (RAM) and the formation of RSC engrams. Activation of RSC in the maze led to a global fluorescence change, which peaked at 2.5–3 hr after entry into the maze (timepoint 3, Figures 1D and S1B) comparable to the time-course reported in a previous study using the same construct [14].

#### Fluorescent Cell Activation Was Task Dependent

**Figure 2:**
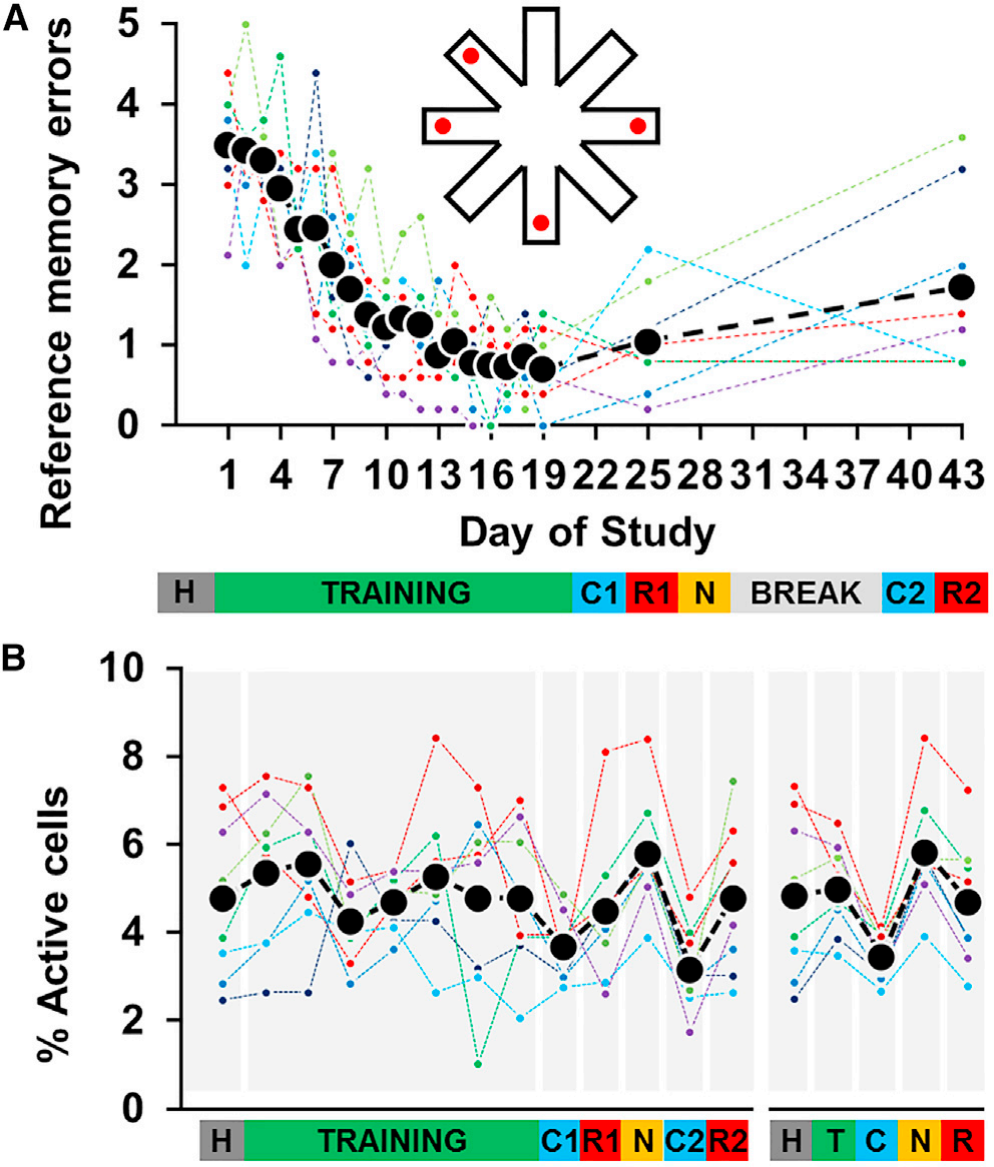
The Pattern of Cell Activity Depended on Experimental Conditions (A) Reference memory errors in the radial-arm maze. An error was defined as entry into a non-baited arm (see inset above graph). Animals trained for 19 days, followed by negative control sessions on days 22 (C1) and 40 (C2), retention tests on days 25 (R1) and 43 (R2), and exposure to a novel environment on day 28 (N). Black symbols represent the mean error score, while colored symbols, the performance of individual animals. H, habituation; C1, C2, negative control sessions; R1, R2, retention sessions; N, novelty condition. (B) The black symbols show the mean percentage of active cells throughout the study, while colored symbols show data from individual animals. On the right-hand side of the graph, days 1–19 were collapsed into a single category of “training” (T), negative control sessions C1 and C2 into a single category of “control” (C), and sessions R1 and R2 into a single category “retention” (R). See also Figure S2.

Mice were imaged repeatedly over a 6-week period, which comprised the acquisition of a reference memory in the RAM over 19 days and a test of long-term memory retention on days 25 and 43. Three control sessions were also included: two negative controls involving placement in the dark and one positive control involving exposure to a novel environment (Figure 2A). Our spatial memory task required the mice to retrieve strawberry milk rewards from four out of eight arms of an RAM (Figure 2A). The positions of the rewards were fixed throughout the study (as was the position of the RAM in the room); hence mice could learn, across sessions, which arms were baited and which were not. The animals showed an 80% ± 11% (mean ± SD) reduction in visits to non-baited arms over the 19 days of training (F(18, 126) = 22.69, p < 0.001). On the other hand, testing after a delay of 6 (R1) and 24 (R2) days revealed a decline in memory (50% and 150% more errors, respectively) which did, nevertheless, remain above naïve levels (with 70% and 49% fewer errors compared to day 1 on days R1 and R2, respectively) (F(2, 14) = 20.34, p < 0.001) (Figure 2A).

Out of 6,229 identified neurons, 27% were considered active (i.e. displayed an evoked time-locked response, see STAR Methods) at least once during the study and were placed in the “active cell pool” (Figure S2). The percentage of cells deemed active on each imaging session was dependent on the experimental condition and was, on average, in the range of 3.2%–5.8% (Figure 2B), similar to levels reported in other *in vivo* immediate-early gene imaging studies [12, 15]. An ANOVA revealed a main effect of condition (F(12, 84) = 2.78, p = 0.003) with novelty displaying the highest percentage of active cells, while the negative control sessions exhibited the lowest overall activity (Figure 2B; training versus negative control and novelty versus negative control, both p < 0.001). Earlier studies in rats have also reported increased *C-fos* expression in RSC following performance of RAM-based spatial tasks [16, 17, 18]. However, while our RAM task increased levels of c-Fos, the percentage of active cells showed no relationship with training session (F(6, 42) = 1.02, p = 0.42). The lack of correspondence between task acquisition and overall c-Fos levels likely reflects divergent activity patterns among various retrosplenial cell types such as head-direction cells, place-like cells, and reward-sensitive cells [9]. Nevertheless, the condition-dependent activity levels highlight the specificity of the c-Fos signal.

#### The Pattern of Cell Activity Depended on Experimental Conditions

**Figure 3:**
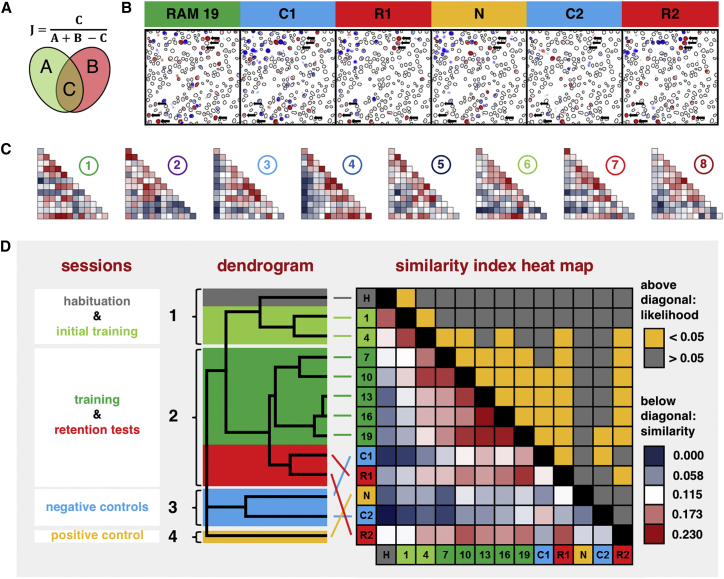
The Pattern of Cell Activity Depended on Experimental Conditions (A) The similarity index *J* was calculated as the ratio of elements in common *C* and the sum of all available unique elements *A + B − C*. (B) A representative example of a population of cells tracked throughout the study. The outlines denote detected cells, while the coloring denotes the signal difference between timepoint 3 and timepoint 1, with red denoting an increase and blue denoting a decrease in fluorescence. The arrows point to putative “engram” cells. (C) Similarity index heat maps generated for individual animals. The arrangement of sessions is the same as in (D). Each square of the heat map displays the similarity index between two experimental sessions. Warm colors highlight high similarity index values, and cool colors highlight low similarity index values. (D) Mean similarity index heat map and corresponding dendrogram. The squares below the diagonal of the heat map represent mean similarity indices, while values above the diagonal represent corresponding (log10) p values with those below (Bonferroni-adjusted) α = 0.05 highlighted in orange. The dendrogram to the left reveals the clustering of conditions based on the mean similarity index values. Four clusters were detected: habituation (H) and days 1 and 4 of RAM training in light green; RAM training days 7–19 with the two retention sessions R1 and R2 in dark green; negative control sessions (C1, C2) in blue; and novelty on its own (N) in orange.

In order to reveal whether neurons activated at random or in a context-dependent manner that would represent an engram, we analyzed the degree of similarity in the pattern of active cells across conditions by using a similarity index [19] (Figures 3A and 3B). Indices were calculated for each pair of sessions and represented graphically in the form of similarity heat maps (Figure 3C). Figure 3D displays the mean similarity indices for the experimental cohort. Acquisition of the spatial task was paralleled by the emergence of a stable pattern of neuronal activation. Similarity scores increased as training progressed, reaching a mean value of 0.23 ± 0.03 between days 13 and 16. The two retention tests also showed a high level of similarity between each other and with the final days of training. In contrast, the control conditions showed low similarity with the other experimental days irrespective of their temporal proximity.

Similarity between conditions can be represented via a dendrogram calculated from the similarity matrix. It clearly reveals the presence of four distinct clusters of conditions (Figure 3D), showing the neuronal activity pattern to differentiate between sets of conditions. The first cluster contained the final day of habituation and RAM sessions 1 and 4, likely reflecting the component of activity associated with the spatial environment itself. The remaining training sessions and retention sessions, during which mice showed evidence of learning, formed cluster number 2. This cluster likely reflects the component of the engram linked to the association of the features of the spatial environment with reward [20]. The two negative-control sessions showed a degree of overlap but were distinct from other sessions (cluster 3) while novelty formed a cluster on its own (cluster 4).

Furthermore, we established that the highest mean similarity indices were unlikely to have arisen due to chance (Figure 3D). The final day of training and the two retention sessions displayed highly significant similarity indices between each other, while novelty failed to produce a significant pattern of overlap with any other condition, even with sessions close in time and despite engaging the largest population of neurons.

#### Engram Stability Predicted the Degree of Memory Retention

**Figure 4:**
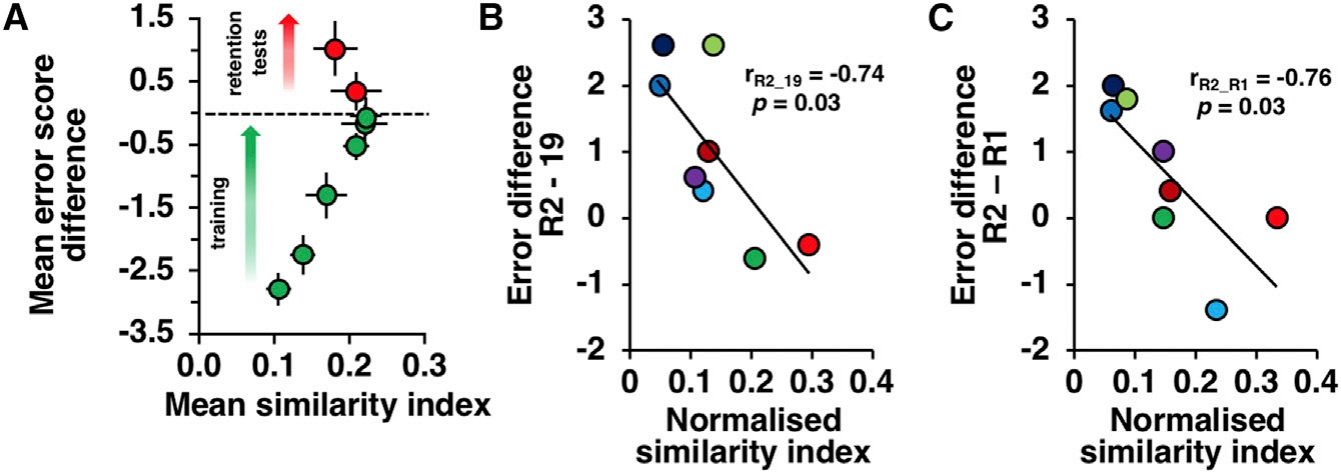
Engram Stability Predicted the Degree of Memory Retention (A) Relationship between mean similarity index and mean difference in error score, between training session 19 and remaining RAM sessions (green symbols); error bars are SEM. As animals become better at the RAM task (error score difference approaching 0), the similarity index increases, whereas the two memory retention sessions (red symbols) show increasing error score difference with decreasing similarity index. (B and C) (B) Relationship between error score difference and normalized similarity index (see STAR Methods) for individual animals plotted for sessions R2 and 19 and, in (C), for sessions R2 and R1. See also Figure S3.

In order to probe the functional importance of *C-fos*-expressing ensembles within the RSC, we examined the relationship between engram stability and behavior. Since training session 19 yielded the lowest error score (Figure 2A), we hypothesized that its pattern of activity was optimal for solving the task. As such, we predicted that during training, neuronal patterns would evolve towards the pattern present on session 19, while on retention sessions, the degree of forgetting would be related to the degree of pattern divergence from that on session 19. Indeed, plotting mean similarity indices between session 19 and all other sessions against the mean difference in reference error score between session 19 and other sessions showed this relationship between forgetting and pattern divergence (Figure 4A). Moreover, the increase of the error score between the final retention session (R2) and training session 19 (24 days earlier) showed a strong, negative relationship with normalized similarity index (see STAR Methods) between those two sessions (r = −0.74, p = 0.034, Figure 4B), as was the case for the first retention session (R1, 18 days earlier) and R2 (r = −0.76, p = 0.029, Figure 4C). The relationship of successful reference memory retention with the sparsity and fidelity of reinstatement of the RSC engram fits well with current views on the requisites of efficient neuronal coding [21, 22].

We also investigated whether calculating percentage cell reactivation produced the same results as the similarity index. The reactivation measure revealed very similar trends but was prone to more noise due to its dependence on absolute active cell numbers. While animals exhibiting higher reactivation scores on the final retention test (R2) were also better performers (Figures S3A and S3B), correlations showed higher variability and did not reach significance. The similarity index, therefore, appears a more sensitive measure for assessing engram stability.

The RSC displays differences in connectivity along its antero-posterior (AP) axis [23, 24], which raises the question of whether the position of ROIs in our study might have influenced our results. While there was a trend for more anterior ROIs to exhibit a higher mean percentage of active cells (Figure S3C; r = 0.59, p = 0.12) (Figure S3C), no relationship between AP position and mean similarity index values was found (r = 0.2, p = 0.64). R2_19 similarity index values showed a positive trend with AP (r = 0.65, p = 0.08), but there was no relationship between R2_R1 similarity index and AP (r = −0.20, p = 0.63). The fact that we did not find a significant correlation between AP position and similarity index is perhaps not surprising given that both anterior and posterior RSC are known to contribute to spatial memory as evidenced by previous lesion and *C-fos* expression studies [16, 25, 26].

#### What Is the Dysgranular RSC Encoding?

The RSC has been proposed to aid the translation between egocentric and allocentric world views during navigation, imagination, and planning for the future [2]. While both granular and dysgranular RSC are engaged during spatial working memory tasks, dysgranular RSC plays a selective role when visual cues are required to perform the task [17]. Moreover, lesions of dysgranular RSC impair rats’ ability to utilize distal visual cues to perform an RAM task [27] or to discriminate between two spatial locations [28]. Dysgranular RSC lesions also disrupt the integration of visual and non-visual cues as shown by an impairment on cross-modal object recognition [29]. This pattern of deficits is in line with the known connectivity of dysgranular RSC: it is densely connected with visual regions, with the posteromedial area forming major source of visual input [8, 30]; and spatial information is provided by the subicular complex [23, 31], while the anterior thalamic nuclei provide both spatial and proprioceptive inputs [32, 33]. As such, the dysgranular RSC forms a nexus for external and internally derived spatial cues.

While the RSC is implicated in online processing of information such as switching and integrating between viewpoints, there is also evidence that it is necessary for storing longer-term memories, consistent with our current findings. There have been reports of retrograde amnesia in patients with damage to the RSC region [34, 35]. Animal studies also highlight the potential importance of the RSC for long-term memory, as post-training RSC lesions impair performance of previously acquired object-based memory tasks [36, 37].

While a number of studies have highlighted a role for the RSC in long-term memory, it is still uncertain whether cortically encoded long-term memories are qualitatively equivalent to hippocampal traces and whether they require hippocampal input for retrieval [38]. Since the hippocampus and the RSC are both important for spatial processing, it could be the case that the RSC engrams described here merely reflect their hippocampal inputs. However, this seems unlikely given the anatomy. Within the RSC, it is the granular subregion that is most densely connected to the hippocampal formation, with dysgranular RSC mainly receiving inputs from the postsubiculum [23]. It is not yet known whether similar engrams are formed in additional areas connected to the RSC, in particular the postsubiculum, prompting the need for future research. Current theories of memory consolidation propose that cortical engrams emerge together with hippocampal engrams but engage in retrieval only following their maturation [15, 39, 40, 41]. This was likely the case in our study, as the RSC spatial representation stabilized with training and was re-instated in this form upon retention tests. This contrasts with current knowledge about hippocampal engrams, which are typically formed much more rapidly [15, 39, 40, 41]. As such, the neuronal ensembles within dysgranular RSC and hippocampus likely function in a complementary manner, with the relative contribution of each varying across time.

### Conclusion

Our study demonstrates the existence of spatial memory engram cells in the mouse RSC. We show that spatial learning is accompanied by the gradual emergence of a context-specific pattern of neuronal activity, which is retained over long periods of time. Furthermore, the stability of the reactivated engram displayed a relationship with the degree of forgetting, providing for the first time direct evidence for the interdependence of spatial memory consolidation and RSC engram formation.

## STAR★Methods

### Key Resources Table

**Table:** 

REAGENT or RESOURCE	SOURCE	IDENTIFIER
**Antibodies**		

Rabbit polyclonal anti-c-fos	Synaptic Systems GmBH	Cat. #226 003
Goat anti-rabbit AlexaFluor-405-IgG	Abcam	ab175652
Rabbit monoclonal anti-NeuN conjugated with Alexa Fluor 647	Abcam	ab190565
Rabbit polyclonal anti-GFP-Alexa Fluor 488	Fisher Scientific	Cat. #A-21311

**Chemicals, Peptides, and Recombinant Proteins**		

Euthathal	Merial Animal Health	N/A (provided by vet)
Dexadreson (dexamethasone)	Intervet UK Ltd	N/A (provided by vet)
Metacam (meloxicam)	Boehringer Ingelheim	N/A (provided by vet)
Chloramphenicol 1%	Martindale Pharmaceuticals Ltd	N/A (provided by vet)
Germolene	Bayer	Available as over the counter product
Lidocaine hydrochloride 2%	Pfizer	N/A (provided by vet)
Vetbond	3M	1469
Super-Bond C&B	Sun Medical	N/A
3 mm small round cover glass	Harvard Apparatus Ltd	64-0720
Sodium chloride	Merck	1064041000
Sodium hydrogen carbonate	Merck	1063291000
Potassium chloride	Merck	1049361000
Sodium dihydrogen phosphate monohydrate	Merck	1063461000
Magnesium chloride hexahydrite	Merck	1058331000
D(+)-glucose	Merck	1083371000
Oxoid™ phosphate buffered saline tablets	Fisher Scientific	BR0014G
Triton-X	Merck	T8787-50ML
Paraformaldehyde	Merck	P6148-1KG
Sucrose	Merck	S9378-1KG
Goat serum	Merck	G9023-5ML
Fluoromount Aqueous Mounting Medium	Merck	F4680-25ML
Strawberry milk	Yazoo, FrieslandCampina	N/A
Vanilla and lemon extracts	FoodCare	N/A

**Experimental Models: Organisms/Strains**		

B6.Cg-Tg(Fos-tTA,Fos-EGFP^∗^)1Mmay/J mouse	The Jackson Laboratory	RRID: IMSR_JAX:018306

**Software and Algorithms**		

Fiji (ImageJ)	[42]	https://fiji.sc
ImageJ StackReg plugin	[43]	http://bigwww.epfl.ch/thevenaz/stackreg/
ImageJ Template Matching plugin	[44]	https://sites.google.com/site/qingzongtseng/template-matching-ij-plugin
ImageJ bUnwarpJ plugin	[45]	https://imagej.net/BUnwarpJ
Custom ImageJ macros	This paper	Mendeley Data: https://doi.org/10.17632/45ts33k2tz.1
R	R Project	Version 3.4.3 https://www.r-project.org/
RStudio	https://www.rstudio.com/	Version 1.1.383
R code	This paper	Mendeley Data: https://doi.org/10.17632/45ts33k2tz.1
SPSS	IBM	Version 23

### Contact for Reagent and Resource Sharing

Further information and requests for data/protocols should be directed to and will be fulfilled by the Lead Contact, Frank Sengpiel (sengpielf@cardiff.ac.uk).

### Experimental Model and Subject Details

#### Subjects

The animals were 8 (7 male) mice carrying a transgene driving the expression of the short half-life (2 hr) enhanced green fluorescent protein (eGFP) from the *C-fos* gene promoter [11] (Jackson Laboratories, #018306). Mice were housed with their littermates under a 14h/10h light-dark cycle and food and water were available ad libitum (except for during behavioural testing, see below). Each cage contained a UFO hamster wheel (Pets At Home; 21035P) similar to that used during the imaging procedure (see below). All animal procedures followed international and institutional standards for the care and use of animals in research and were approved by the Animal Welfare and Ethical Review Body at Cardiff University and by the UK Home Office under project licence 30/3309.

### Method Details

#### Surgery

Craniotomies were performed at 8-10 weeks of age as previously described [46], with round (3 mm radius) glass coverslips (Harvard Apparatus Ltd) placed over the dorsal (dysgranular) retrosplenial cortex (Figures 1A–1C). Mice were anaesthetised with isoflurane (1.5%–2%, 100% O2) and the body temperature was maintained with a heating pad. Each animal received an intramuscular injection of dexamethasone (30 μL; Intervet UK Ltd) to reduce cortical swelling and a subcutaneous injection of an analgesic (50 μL of 10% solution of Meloxicam; Boehringer Ingelheim); the eyes were protected with an eye ointment (Chloramphenicol, Martindale Pharmaceuticals Ltd). After placement in ear-bars, the hair on the scalp was smoothed down with a layer of Germolene gel (Bayer) and sterilised with the application of iodine, followed by a rinse with 70% ethanol. A local, fast-acting analgesic (lidocaine 2%; Pfizer) was then injected under the scalp and the skin resected to expose the skull. The skin around the incision was sealed with tissue glue (Vetbond; 3M) and dried with 70% ethanol. A layer of dental cement (Super-Bond C&B; Sun Medical) was applied and a custom-made aluminium head-plate was affixed to the top of the skull.

The cement was left to dry for 20 min after which the animal was transferred from ear-bars to a head-holder device. Excess cement was drilled off and a craniotomy was performed to reveal the cortex. The brain was rinsed several times with standard ice-cold artificial cerebrospinal fluid (ACSF; 119 mM sodium chloride, 26.2 mM sodium hydrogen carbonate, 2.5 mM potassium chloride, 1 mM sodium dihydrogen phosphate monohydrate, 1.3 mM magnesium chloride hexahydrite, 10 mM glucose; all from Merck) and a glass window was inserted to replace the missing skull-flap. Excess ACSF was removed and the edges of the cranial window were sealed with tissue glue, dried, and secured with dental cement after which the animal was transferred to an incubator to recover. Imaging procedures began after 14 days to allow for the clearing of the windows and recovery.

#### Radial-arm maze apparatus

The central platform of the maze was a regular wooden octagon with 8 wooden arms attached, each measuring 9 by 36 cm. The walls of the arms were made of clear Perspex panels 17 cm high. The joining of the maze was made of black 3D-printed components. Each arm was separated from the central platform by an upside-down guillotine door (made of clear Perspex) pulled close via strings threaded through an arrangement of pulleys mounted to the underside of the platform. In this way, the experimenter could control access to the individual arms. At the end of each arm there was mounted a black 3D-printed element containing a 50 μL reward well. The maze was elevated at 100 cm above ground and evenly illuminated by ambient light. The maze was situated in the corner of a large room with a number of visual features that could be used to orient and navigate, for example, cupboards, a fridge as well as an intrinsic imaging set-up; the position of the maze within the room remained fixed throughout the study.

#### Behavioural training

##### Behavioural timeline

Animals were habituated to the experimental procedures in four stages: habituation to handling, habituation to head-fixation, habituation to imaging, and finally, habituation to the RAM. Training in the RAM lasted 19 days. Two negative control sessions were carried out on days 22 and 40 and a positive control session, novel environment exploration, was carried out on day 28. The retention of memory acquired during RAM training was tested on days 25 and 43. The mice were imaged on the last day of maze habituation, seven out of the 19 RAM sessions (days 1, 4, 7, 10, 13, 16, 19), the two negative (days 22 and 40) and positive (day 28) control sessions and on the two retrieval sessions (days 25 and 43). Animals were also subject to the habituation to imaging procedure for two days prior to any imaging session (including during the RAM training stage). Finally, the mice were sacrificed following imaging on day 43.

##### Habituation

The first stage of habituation lasted five days and simply involved handling the animals during daily post-operative care. Over the next six days, mice were gradually habituated to head-fixation by being immobilised for short intervals (1-5 min) with the use of forceps. Following this, the mice were introduced to the 2-photon imaging set-up. Mice were head-fixed and placed on top of a UFO hamster wheel (mounted beneath the microscope objective), in the darkness, for 10 min. The same procedure was repeated on the three following days with running durations of 45 min, 60 min and 60 min. Immediately before and after running on the wheel, each mouse was placed in its individual ‘experimental cage’ in the dark for 30 min. The ‘experimental cage’ was a standard small rodent cage with standard bedding but without access to food or water.

Next, the mice were habituated to the RAM where they were allowed to freely explore for 30 min (all arms open). While in the maze, animals were also exposed to strawberry milk (Yazoo, FrieslandCampina) for the first time. On the first day of maze habituation, strawberry milk droplets were evenly scattered around the maze and inside the reward wells at the end of each arm. On subsequent days, the number of strawberry milk droplets was gradually reduced such that on the final habituation day, rewards were only present inside and immediately around the reward wells. Following the three days of maze habituation, all mice were given two further days of exposure to imaging procedures.

The animals’ liquid intake was reduced at this stage of the study. Each mouse was allowed as many strawberry milk droplets as were available on a given day and was further supplemented with water up to a volume of 1 mL of total daily liquid intake. Water was made available only after all experimental procedures on a given day were completed.

#### RAM training

Mice were subsequently trained on a reference memory task in the RAM. The animals’ task was to retrieve four strawberry milk rewards located in reward wells at the end of four out of the eight arms of the RAM. The rewarded arms (arm numbers 2, 3, 4 and 8) were consistent across training and across animals. Each testing day consisted of five trials. At the start of each trial, the animal was placed under a cardboard box on the central platform of the maze. The box was lifted and after a delay of 10 s, the arm doors were open. The animal was allowed to visit any of the arms to look for rewards. Arm visits were manually recorded for each trial. Upon return to the central platform, the guillotine doors were closed again for 10 s until the next run began. In total, animals were allowed either up to eight attempts or up to 5 min to retrieve all four rewards. The trial also ended if the animal successfully retrieved all four rewards before eight attempts had been made. The mice were confined to the central platform underneath a cardboard box in-between the trials while the experimenter wiped the maze with 70% ethanol and replenished the consumed rewards. In total, an experimental session lasted up to 30 min (5 x 5 min for each trial plus 5 min for cleaning/replenishing rewards). The mice were placed in their ‘experimental cage’ in the dark for 30 min prior to testing and returned to the ‘experimental cage’ after testing was over. Training on the RAM task lasted for 19 consecutive days.

#### Negative control condition (C1 and C2)

The mice underwent two negative control sessions (on day 22 and day 40). Under the negative control condition, mice first spent 30 min in the ‘experimental cage’ as they would before the RAM testing sessions. They were then briefly handled and returned to the ‘experimental cage’ together with a plastic weighing boat filled with 20 droplets of strawberry milk (equivalent to the maximum reward available on testing days) for a further 30 min in the dark. Finally, the weighing boat was removed, the mice handled briefly again and returned to the ‘experimental cage’.

#### Positive control condition (novel environment exploration, N)

For the novel environment exposure session (day 28), mice first spent 30 min in the ‘experimental cage’ in the dark, as under the testing and negative control conditions. After that, they were transferred to a white Styrofoam box equipped with various visual, tactile, and odorant stimuli. The interior dimensions of the box were 26 by 31 by 26 cm. The box contained two levels: the floor of the box and a raised platform accessible via a ramp. The floor and the walls of the box were covered with black and white geometric patterns. The box also contained small weighing boats (on its floor and attached to the wall), a plastic string suspended between two of the walls, two glass electrode filaments mounted against the walls and two dental cotton rolls (one on the floor, one on the wall) infused with vanilla and lemon extracts (FoodCare) respectively. Finally, the box also contained four reward wells moved from the RAM: three on the ground level and one on the level of the raised platform. Mice were placed in the box five times for 5 min, mimicking the five test trials on RAM sessions, and were provided with strawberry milk in the four reward wells on each run. In-between runs the mice were confined under a cardboard box. Following the procedure, animals were returned to their ‘experimental cages’.

#### 2-photon imaging

##### Apparatus

Mice were imaged under a custom-built 2-photon microscope (MOM, Sutter Instruments, USA) with a Ti:Sapphire laser (MaiTai DeepSee, Newport SpectraPhysics, UK) using a 20×, 1.00 NA Olympus (N20X-PFH) water immersion objective. During imaging, mice were placed on top of a UFO hamster wheel and head-fixed. The design of the wheel allowed the mice to decide whether they wanted to remain stationary or to move and thus helped to minimise stress. Imaging was performed in the dark with the set-up tightly covered with a black opaque curtain. The laser was tuned to 940 nm and maintained within the power range of 35.0-35.3 mW.

##### Regions of interest

The regions of interest (ROIs) for imaging were chosen based on the appearance of the cortex under brightfield illumination and then refined based on the visibility of fluorescence under the 2-photon microscope. Following perfusion on day 43 of the study, another set of images was taken to assess the position of the cranial window in relation to gross anatomical landmarks. This allowed for the subsequent alignment of the 2-photon, brightfield, and post-mortem images and made it possible to mark the precise position of the imaging ROI for each animal.

##### Imaging timeline

The timeline consisted of the 4 following stages: (1) 30 min in the dark in the ‘experimental cage’; (2) RAM/control session lasting up to 30 min; (3) return to the ‘experimental cage’ until 60 min from the beginning of stage 2 had elapsed; (4) placement under the 2-photon microscope and the alignment of the region of interest with reference; (5) imaging commenced 90 min from the onset of stage 2. Fluorescent images were taken at 15 min intervals over the space of 2 hr (90 min, 105 min, 120 min, 135 min, 150 min, 165 min, 180 min, 195 min). Following imaging, mice were returned to their cages and given water if they have not already consumed 1 mL of liquid that day.

##### Imaging parameters

At each imaging timepoint, a stack of 70 16-bit images was acquired. The stack was of the following dimensions: 400×400 μm wide (corresponding to 1024×1024 pixels) and 140 μm deep (70 pixels). The z-position of the first image in the stack lay between 100-130 μm under the pial surface, corresponding to the outer border of cortical layer 2. The acquisition of each stack lasted around 8 min and mice were left undisturbed in-between the imaging timepoints unless the objective water required topping-up.

##### Immunohistochemical detection of c-Fos and eGFP

Immediately after imaging on day 43, mice were terminally anaesthetised with a 1 mL intraperitoneal injection of sodium pentobarbitone (Euthathal; Merial Animal Health). They were then intracardially perfused with a solution of 1% paraformaldehyde/PBS (Merck) and stored in the fixative at 4^o^C until cut. Brains were incubated in 25% sucrose (Merck;) in PBS overnight prior to cutting and sliced in the coronal plane at 30 μm with a sliding microtome (Jung SM; Leica), followed by cryopreservation in a glycol/sucrose solution at -20^o^C.

For each animal, a 1:4 series of brain slices was processed for the detection of c-Fos, eGFP and NeuN. The slices were first thoroughly rinsed in PBS solution (Oxoid)(first overnight and then 4×15 min) and then incubated in blocking solution (PBST: 0.3% Triton-X in PBS (Fisher Scientific UK Ltd, UK) and 2% normal goat serum (Merck)) for 1 hr. After that, they were transferred into the first primary antibody cocktail containing a 1:5,000 concentration of rabbit anti-c-Fos polyclonal antibody (Synaptic Systems GmBH) in the blocking solution at 4^o^C for 72 hr. The slices were then washed 4×15 min in PBST and incubated in a solution of 1:2,500 goat anti-rabbit AlexaFluor-405-IgG antibody (Abcam) in the blocking solution for 2 hr at room temperature. The slices were then again washed 4×15 min in PBST and incubated in the second primary antibody cocktail. This was a solution of 1:2,500 rabbit anti-NeuN-AlexaFluor647 (Abcam) and 1:2,500 rabbit anti-GFP-AlexaFluor488 (Fisher Scientific UK Ltd) monoclonal antibodies in the blocking solution. The incubation was again at 4^o^C and over 72 hr, after which the slices were washed 4×15 min in PBST and mounted on gelatine-coated glass slides (Fisher Scientific UK Ltd; 12312148). The slices were protected with an aqueous mountant solution (Fluoromount; Merck).

Brain slices were imaged under a Zeiss LSM 880 upright confocal microscope with Airyscan equipped with a Plan Apochromat 20×/0.8 air objective (Zeiss). NeuN, c-Fos and eGFP were detected with the HeNe 594 nm, Diode 405 nm and Argon multi-line 458/488/514 nm laser lines, respectively. The wavelength filtering was set to default software values indicated for the imaged fluorophores and image size was 650 x 650 x 16 μm (2048 x 2048 x 7 pixels). For each animal, two z-planes were selected for analysis and merged using maximum intensity projection in Fiji [42]. Then, neurons were detected by thresholding the red channel (NeuN; auto-threshold function) and the obtained masks were used to measure pixel intensity values in the green (eGFP) and blue (c-Fos) channels. Finally, for each animal, c-Fos- and eGFP-positive neurons were detected by applying a threshold (mean + 1.5 x standard deviation of the measured values) ensuring only the brightest cells were detected in each channel (see *in vivo* cell detection below).

### Quantification and Statistical Analysis

#### RAM performance

Acquisition of the spatial task was assayed by tracking the mean number of errors per session. Total errors were split into two categories to reveal the working-memory (WM) and reference memory (RM) components of the task. A WM error was defined as re-entry into an arm previously visited during the trial whilst an RM error occurred when the animal visited a non-baited arm (i.e. arms 1, 4, 5 and 7).

#### Pre-processing of 2-photon image stacks

The pre-processing of image stacks was carried out in a semi-automated way in Fiji. The xy drift along the z axis was corrected with the StackReg plugin (Rigid Registration [43]) for each stack and images were also lightly filtered (Kuwahara filter, sampling = 3; followed by median, radius = 2). The next step involved precise manual alignment of stacks across days in the z axis as well as a coarse alignment in the xy plane. All stacks were then truncated to only include the 60 best overlapping z planes. This was followed by the reduction of the z dimensionality from 60 images to 12 images per stack. In this way, each image in a stack now corresponded to 10 μm in the z axis. The reduction in dimensionality was carried out by first filtering the images using the built-in 3D Gaussian filter (x, y, z = 2) and then obtaining maximum intensity projection images for every 5 images in the stack.

The next stage involved pooling together all corresponding z planes from stacks across the days and timepoints and creating 12 superstacks of 104 images (8 timepoints × 13 sessions) for each animal. These were then aligned in the xy plane using the StackReg (Rigid Registration) and Template Matching [44] (default parameters) plugins. The next step utilised the bUnwarpJ plugin [45] to correct for non-linear distortions in the xy plane. First, the histograms of all images in the superstack were equalised by using the Bleach Correction (Histogram Matching) option from the Adjust menu. Then, from each superstack, a reference image was created by running a maximum intensity projection and used as template for the bUnwarpJ transformations. The transformations were run iteratively starting with coarse parameters (Registration mode: Accurate; Initial Deformation: Coarse; Final Deformation: Fine, remaining parameters: default) and progressing to finer parameters (Registration mode: Accurate; Initial Deformation: Fine; Final Deformation: Very Fine, remaining parameters: default). The resulting images were then manually cropped to only include a ROI region with non-NaN pixels and maximum intensity projections of these images were combined into a stack for each case. These were then aligned using the Template Matching plugin and cropped to exclude NaN pixels. The x and y translation values from the Template Matching plugin were manually inputted to translate stacks 1-12 for each animal which were further cropped to display the same xy dimensions across all 12 stacks.

#### Detection of fluorescent cells

The 12 cropped and aligned superstacks were then combined using the montaging option. For each animal, a montage of all images from across all stack levels and conditions was created such that it consisted of 104 rows (temporal order of images) and 12 columns (the 12 z levels). These were then turned into a single stack of 1248 images and their histograms were equalised across the stack (Adjust->Bleach Correction->Histogram Matching). The stack was then converted into a new stack of 104 images, each containing the 12 z levels in a row and saved for further analysis (measurement stack). A standard deviation image projection was carried out on the measurement stack.

The standard deviation projection was used for the manual masking of fluorescent cells. For each identified neuron, the z plane with the largest cross-section was sought and a round (7 μm radius) ROI mask was assigned to it at that position. The temporal dimensionality of the measurement stack was then reduced. For each pair of adjacent timepoints (e.g. 90 min and 105 min, 120 min and 135 min), a mean stack was created and filtered with a Gaussian filter (x, y = 2). From this point onward, the following timepoints were used: (1) 90 min to 120 min; (2) 120 min to 150 min; (3) 50 min to 180 min; and (4) 180 min to 210 min. The ROI masks were then applied throughout the stack so that a mean grey value per timepoint and condition could be measured.

#### Timecourse of fluorescence change and percentage active cells

Cell fluorescence values were measured on an 8-bit scale. The timecourse of fluorescence changes was calculated by taking the mean for each animal, at each timepoint, across all RAM sessions. The values were then standardised so that timepoint 1 would represent a mean value of 0 across all cases (by dividing each timepoint by the value at timepoint 1 and subtracting from all timepoints the mean value obtained for timepoint 1; Figure S1B).

Then, for each cell, the difference in fluorescence intensity was taken between the third and the first imaging timepoints (as timepoint 3 presented the greatest fluorescence increase) and plotted on a frequency histogram. The resulting frequency distribution of the calculated values assumed a largely symmetrical double-exponential shape with a mode around 0 (Figure S2) and a longer positive tail, indicating that the vast majority of cells did not see large departures from timepoint 1 values while a small proportion of cells saw very high fluorescence increases. To define ‘active cells’, the fluorescence difference values calculated across sessions were pooled to derive a single standard deviation measure for each animal. A cell was considered ‘active’ if its change in fluorescence value was positive and exceeded the mean by 1.5 times the standard deviation of the pooled distribution of fluorescence change for each animal (similar to another study [10]. This threshold was chosen to restrict ‘active cells’ to approximately 5% of all observed values, therefore analogous to carrying out a one-tailed z-test with an alpha level of 5%. The ‘percentage’ active cells index was calculated by dividing the number of ‘active’ cells on a given session by the total number of detected cells for each animal and multiplying it by 100. For certain analyses, values were collapsed across a number of sessions by taking an arithmetic mean (such as for the RAM days 1-19, which were collapsed into a single experimental block: ‘training’, see Figure 2B).

#### The calculation of the similarity index and hierarchical clustering

The similarity index (Jaccard [19]; ***J***) was calculated for each pair of conditions based on the following equation: J=c/a+b−c, where ***c*** represents the number of cells which ‘co-activated’ between session n and n+1, ***a*** represents the number of ‘active’ cells in session n and ***b*** represents the number of ‘active’ cells in session n+1. Heat maps for individual animals were created in Microsoft Excel by applying conditional formatting. A compound similarity index heat map was calculated by taking the arithmetic means of similarity index values across cases. A dendrogram revealing the clustering of conditions was derived by using the ‘Hierarchical Cluster Analysis’ function (‘between-group linkage’).

#### Calculation of similarity index p values

The probabilities of the occurrence of similarity index values by chance were calculated in R. The probability of the occurrence of a similarity index value (***P***_***J***_) for the set of inputs ***a*** (number of cells ‘active’ on session n), ***b*** (number of cells ‘active’ on session n+1), ***c*** (number of cells ‘co-active’ on sessions n and n+1) and ***t*** (number of cells in the ‘active cell pool’) was derived from the following equation: $P_{J}=\left(C_{c}^{a}*\;C_{b-c}^{t-a}\right)/C_{b}^{t}$, where *a*, *b*, and *t* are fixed parameters associated with each observed similarity index and ***c*** is a hypothetical value sampled from the set of all its possible values (between 0 and min(***a***, ***b***)). $C_{c}^{a}$ represents all possible unique arrangements of overlapping cells among all cells which activated on session n. $C_{b-c}^{t-a}$ represents all possible unique arrangements of active cells on session n+1 which did not overlap with session n. $C_{b}^{t}$ represents all possible unique arrangements of active cells on session n+1 unrestricted by the overlap term ***c***. The obtained values were then expressed in the form of a probability distribution function and the probability of the observed similarity index being greater than the chance similarity index values was calculated. This was achieved by summing the probabilities for hypothetical similarity indices ≥ the experimental similarity index (thereby calculating the probability of the similarity index being at least as high as that observed experimentally). The calculated p value therefore represents the probability of the observed similarity index being greater than all the possible similarity indices for a given *a*, *b*, and *t*. Finally, compound p values across all animals for each pair of conditions were calculated by employing Fisher’s method in R (‘metap’ package) and the α level was adjusted according to the Bonferroni method of family-wise error correction accounting for the 78 tests run in parallel (α’ = α/78).

#### Relationship between similarity index and behavioural scores

Behavioural performance was assessed in reference to the final RAM training session (session 19) by calculating the error score difference between that session and any other session in chronological order. Consequently, values during training are negative and values obtained for sessions R1 and R2 are positive. The mean error difference scores were then plotted against mean similarity index values between session 19 and other RAM sessions.

Performance of individual animals on retention test R2 was assessed by plotting error score difference between session R2 and sessions R1 and 19 against their respective, normalised similarity index values. The normalisation was performed to account for the non-specific component of the similarity index as well as differences in absolute similarity index value ranges across cases (related to the quality of craniotomies). It was conducted by dividing the R2_R1 and R2_19 indices by the R2_C1 index for each animal (followed by division by a single value for all data points, calculated as mean normalised index/mean non-normalised index, to constrain normalised indices within the range of 0 and 1).

#### Relationship between % reactivation scores and behavioural scores

Reactivation scores were calculated as the ratio of cells which activated on retention test session R2 that were also active on training session 19 or retention test session R1, and the number of all cells which activated on training session 19 or retention test session R1, respectively, times 100. These scores were further normalised by division to the predicted chance reactivation scores calculated as the number of cells on reference session (19 or R1)/ active cell pool ^∗^ active cells on session R2/ active cell pool [39].

#### Statistical analyses and figures

Data were analysed for the same 8 animals and for all sessions throughout the study (except for histological detection of eGFP expression, Figure S1A, where 5 animals were used). The comparisons of behavioral scores, fluorescence values, and the percentage of ‘active’ cells were performed in SPSS (IBM) using the repeated-measures general linear model function while similarity index probability was modelled in R (described above). The correlations between error score difference and normalised similarity indices/reactivation scores as well as antero-posterior position and mean percent cell activation were also performed in SPSS (Pearson product moment). Violations of sphericity were corrected for with the Greenhouse-Geisser adjustment of degrees of freedom. The family-wise error for multiple comparisons was corrected for by employing the Bonferroni adjustment of p values. For all tests, the alpha level was set at *p* < 0.05.

### Data and Software Availability

The accession number for the software scripts and data reported in this paper is: https://doi.org/10.17632/45ts33k2tz.1

## Supplemental Information

Document S1. Figures S1–S3

Document S2. Article plus Supplemental Information
