# Supplementary material for: Spatial Memory Engram in the Mouse Retrosplenial Cortex
Source: Curr Biol. 2018 Jun 18;28(12):1975–1980.e6. doi: 10.1016/j.cub.2018.05.002 (PMC6013279; doi:10.1016/j.cub.2018.05.002)
Supplement: Document S1. Figures S1–S3 [file mmc1.pdf]

Current Biology, Volume *28*

Supplemental Information

Spatial Memory Engram
in the Mouse Retrosplenial Cortex

Michal M. Milczarek, Seralynne D. Vann, and Frank Sengpiel

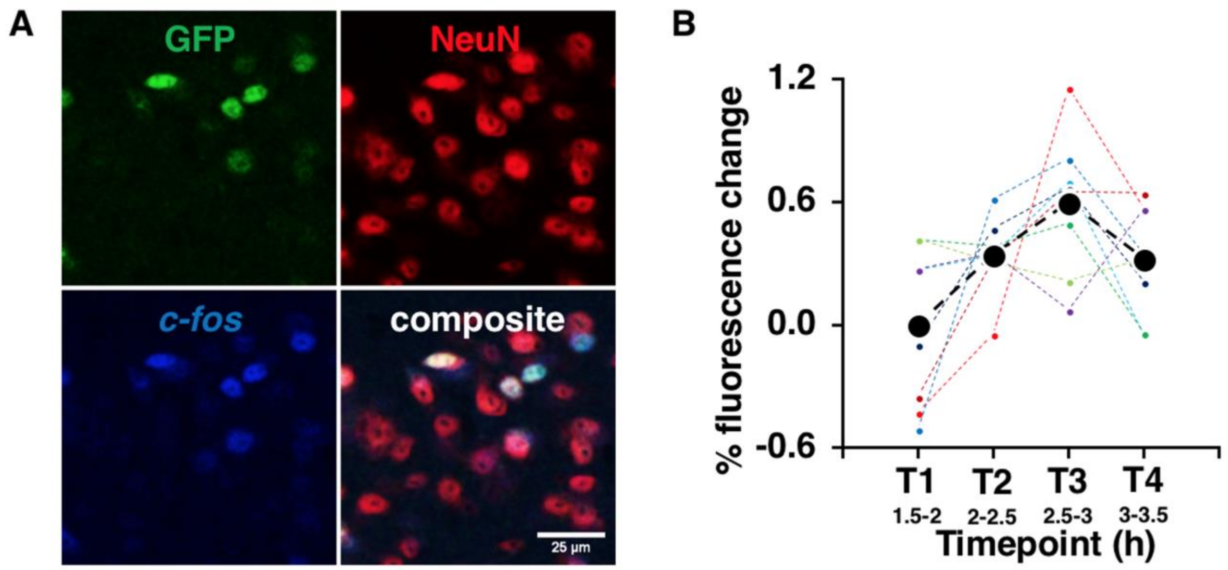

Figure S1. Confirmation of the suitability of the mouse model for task-evoked c-Fos imaging. Related to Figure 1.

(A) Immunohistochemical detection of eGFP and c-Fos co-localisation in layer 2/3 of the dysgranular retrosplenial cortex. eGFP cells (green) constituted 91.54±13.46% of all c-Fos (blue) cells and 7.63±1.08% of all NeuN cells (red). Images were taken from five mice, following the final memory retention imaging session (session 43). (B) The timecourse of mean standardised fluorescence change following exposure to the RAM. Coloured symbols and connecting lines indicate individual animals while mean values are presented in black. The temporal intervals are the same as in Figure 1D. The signal peaked at timepoint 3 (2.5-3 hours from entry into the maze)($F(3, 21) = 3.83$, $p = 0.025$; timepoint 1 vs timepoint 3: $p = 0.04$). A similar timecourse of fluorescence was reported before [S1].

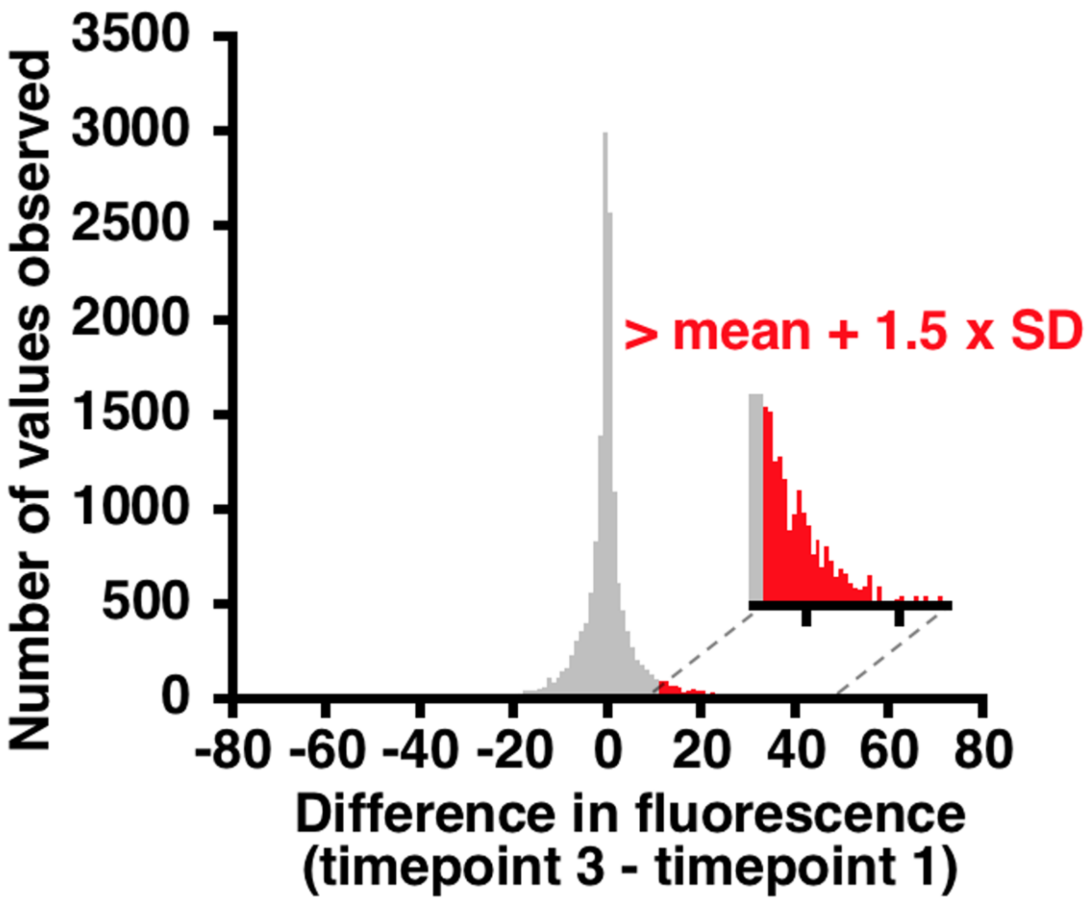

Figure S2. Designation of active cells. Related to Figure 2.

A histogram of differences in fluorescence between timepoint 3 and timepoint 1 calculated across all 13 imaging sessions for one of the experimental animals. Red bars indicate values above the threshold of mean + 1.5 x standard deviation (SD).

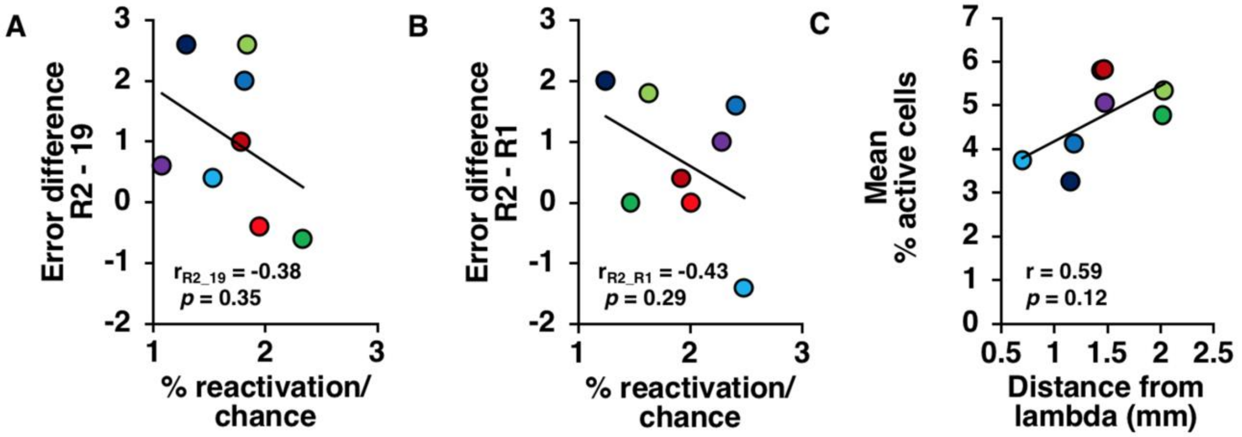

Figure S3. (A-B) Percent reactivation scores versus behavioural performance and (C) the influence of antero-posterior position on observed percent cell activation levels. Related to Figure 4.

Supplemental References

S1. Kim, Y., Venkataraju, K.U., Pradhan, K., Mende, C., Taranda, J., and Turaga, S.C. (2015). Mapping Social Behavior-Induced Brain Activation at Cellular Resolution in the Mouse. Cell Reports 10, 292–305.
